# Effects of tendon injury on uninjured regional tendons in the distal limb: An *in-vivo* study using an ovine tendinopathy model

**DOI:** 10.1371/journal.pone.0215830

**Published:** 2019-04-23

**Authors:** Albert S. Tsang, Andrew J. Dart, Sara A. Biasutti, Leo B. Jeffcott, Margaret M. Smith, Christopher B. Little

**Affiliations:** 1 Research and Clinical Training Unit, University Veterinary Teaching Hospital, Sydney School of Veterinary Science, Faculty of Science, University of Sydney, Camden, Australia; 2 Raymond Purves Bone and Joint Research Laboratories, The Kolling Institute, Sydney Medical School, University of Sydney, Sydney, Australia; 3 The Institute of Bone and Joint Research, Royal North Shore Hospital, Sydney, Australia; University of Pittsburgh, UNITED STATES

## Abstract

Following injury to a tendon little is known about potential for pathology to develop in other regional tendons from overloading or altered function. The aim of this study was to investigate the gene expression and histopathological changes that occur 1) within the deep digital flexor tendon (DDFT) after injury to the superficial digital flexor tendon (SDFT) and 2) within the flexor tendons (SDFT and DDFT) after injury to the extensor tendons. Merino wethers [*Ovis aries*] (n = 18) were divided into three equal groups and underwent either partial transection of the SDFT, complete transection of the extensor tendons or were left as non-operated controls. Tendons were harvested and sampled regionally for gene expression (real time PCR) and histologic analysis eight weeks after surgery. Transection of the SDFT resulted in increased expression of collagen III, versican, biglycan, lumican and MMP1 (P<0.026 for all genes) within the DDFT. There was no effect of transecting the extensor tendons on the expression of any gene tested in either the SDFT or the DDFT. The DDFT had elevated histopathology scores induced by transection of the SDFT, eight weeks previously. There were minimal histological differences in either the SDFT or DDFT after transection of the extensor tendons. Transection of the SDFT results in a mild, subclinical tendinopathy within the DDFT with potential implications on treatment and rehabilitation of SDFT injuries. Injury to the extensor tendons has minimal measured effect on the SDFT or DDFT.

## Introduction

Tendon injuries are a common problem in human and equine athletes [1–3]. They are the most common form of musculoskeletal injury in the horse and have been reported to account for up to 46% of all musculoskeletal injuries in athletic horses [4]. These injuries can be split into two main groups distinguished by clinical presentation and aetiology, into either traumatic lacerations or strain-related injuries.

Horses have evolved to be a predominately flight animal [5], as such, when startled horses display an instinctive response to flee from danger. Although horses have been domesticated for several thousands of years [5], this reaction has not been eliminated and, when housed in confined spaces, horses often injure themselves when startled. Due to the limited amount of soft tissue on the distal limb of horses, traumatic lacerations often involve the underlying tendons and bones [6, 7].

Athletic horses are prone to developing tendinopathies, of which, the superficial digital flexor tendon (SDFT) is by far the most commonly affected tendon [8–11]. In some Thoroughbred racing populations, the SDFT has been implicated in up to 93% of soft tissue injuries [11].

Regardless of the cause of injury, outcomes are often suboptimal and there are significant rates of re-injury or injury in the contralateral limb [12, 13]. The pathophysiology of tendinopathy remains unclear however the current consensus theory involves accumulation of microtrauma coupled with an ineffective healing response [14, 15]. Histologic changes observed in both pathologic and healing tendon includes proteoglycan accumulation, collagen fibre disruption, increased blood vessel infiltration, increased cellularity and cell rounding [16–18]. These histologic changes are accompanied by widespread alterations in gene expression. Recently it has been shown that these changes occur throughout the entire tendon not just localised at the site of injury [18]. These widespread changes have been postulated to contribute to the risk of re-injury at a site adjacent to the original lesion.

Throughout its evolution, there has been a reduction in the number of digits, muscles and tendons in the equine limb [5]. As a result of this reduction, several of the soft tissues structures have taken on a number of different roles. The SDFT and suspensory ligament (SL) have evolved into elastic, energy-storing tendons that limit the ability of the metacarpophalangeal/metatarsophalangeal joints to hyperextend as well as conserving energy to increase the efficiency of locomotion [19, 20]. The primary function of the extensor tendons involves placement of the digits during the swing and stance phases of locomotion [19, 21]. As a result, several complex interactions have developed within and between specific tendon groups. Alterations or total disruption to the function of these tendons could have potential effects on the health and function of other tendons within the equine distal limb.

Several models have been used in equids to investigate the intratendinous environment and assess the effects of different therapies [22–28]. However, these models only assess the tissues adjacent to the affected tendon and did not assess the entire length of the tendons. The authors have developed a number of surgical animal models of tendinopathy, in both sheep and horses, where the tendons can be sampled regionally in order to study the histopathologic and gene expression changes that occur throughout the tendon [1, 18, 29]. Horses and sheep display similar kinematic properties regarding weight distribution between front limbs and hindlimbs and gaits of locomotion as well as displaying almost identical anatomy of the tendons and ligaments within the distal limb [30]. Features of clinical tendinopathy in horses, such as changes in histology and gene expression, have been observed in surgical models of tendon injury in both horses and sheep [1, 18, 29]. Being cheaper to purchase, more easily operated without specialist surgical facilities required for horses, and easier to manage, sheep are ideal candidates to model the effects of injury in tendon.

The aim of this study was to identify any changes in gene expression and histopathology in the flexor tendons following injury to adjacent tendons in an ovine model of surgically-induced tendinopathy. More specifically, we hypothesized that partial transection of the SDFT would result in tendinopathic changes within the deep digital flexor tendon (DDFT). We further hypothesized that complete transection of the extensor tendons would not affect the SDFT or the DDFT.

## Materials and methods

### Ethics statement

All experimental animal protocols were approved by the Animal Ethics Committee of the University of Sydney (AEC no. 2014/642) in line with the Animal Research Act (1985) of New South Wales, Australia. Animal experiments were performed in accordance with the eighth edition of the Australian Code for the Care and Use of Animals for Scientific Purpose (2013). Anaesthesia for surgical procedures was induced with a combination of ketamine and diazepam and anaesthesia maintained with inhalational isoflurane in 100% oxygen. Euthanasia was performed with an overdose of intravenous pentobarbitone.

### Animal allocation and treatment

Eighteen two-year old merino wethers [*Ovis aries*], sourced from a commercial farm, with no history of illness or injury, were used in this study. Sheep were maintained on pasture under normal grazing conditions and supplemented with lucerne hay as required. Sheep were randomly allocated to three groups with six sheep in each group. A sample size of six has previously been shown to be sufficient to detect a two-fold change in gene expression with 80–98% power depending on the gene [29]. Sheep in the first group underwent partial transection of the SDFT in one forelimb. The second group underwent complete transection of the common digital extensor tendon (CDET), medial branch of the common digital extensor tendon (MDET) and the lateral digital extensor tendon (LDET) in one forelimb. Limb distribution was randomly allocated prior to surgery. The sheep in the remaining group were left as non-operated controls and did not undergo any procedures.

#### Pre-operative procedures

Sheep were pre-medicated with diazepam (0.2mg/kg IV) [Ilium Diazepam, Troy Laboratories Pty Ltd, NSW, Australia] prior to placement of an intravenous catheter [14G BD Angiocath, BD Medical, NSW, Australia] into the jugular vein. A combination of diazepam (0.1mg/kg IV) [Ilium Diazepam, Troy Laboratories Pty Ltd, NSW, Australia] and ketamine hydrochloride (5mg/kg IV) [Ilium Ketamil, Troy Laboratories Pty Ltd, NSW, Australia] was administered for anaesthetic induction. A cuffed, endotracheal tube was placed and sheep were maintained on isoflurane in 100% oxygen for the duration of the procedure. Ceftiofur sodium (5mg/kg IV BID) [Accent, Zamira Life Sciences, QLD, Australia] was administered for antibiotic prophylaxis and methadone (0.1mg/kg IV) [Ilium Methadone, Troy Laboratories Pty Ltd, NSW, Australia] and flunixin meglumine (1.1mg/kg IV) [Ilium Flunixil, Troy Laboratories Pty Ltd, NSW, Australia] were administered for analgesia after induction of anaesthesia.

#### Extensor tendon surgery

The entire metacarpus of one randomly assigned forelimb was clipped and the midpoint of the metacarpus was marked with indelible ink to determine the level of tendon transection. Following aseptic preparation, a 5cm longitudinal skin incision was made on the dorsal aspect of the metacarpus. All three extensor tendons were isolated and completely transected with a scalpel blade. The skin incision was closed with absorbable monofilament sutures [3–0 PDS II (Polydioxanone), Ethicon LLC, Puerto Rico, USA], and a light bandage, consisting of sterile swabs [BSN Medical (Aust.) Pty Ltd, VIC, Australia] and cohesive bandage [Henry Schein Inc, NY, USA], was applied to the limb.

#### Flexor tendon surgery

**Fig 1:**
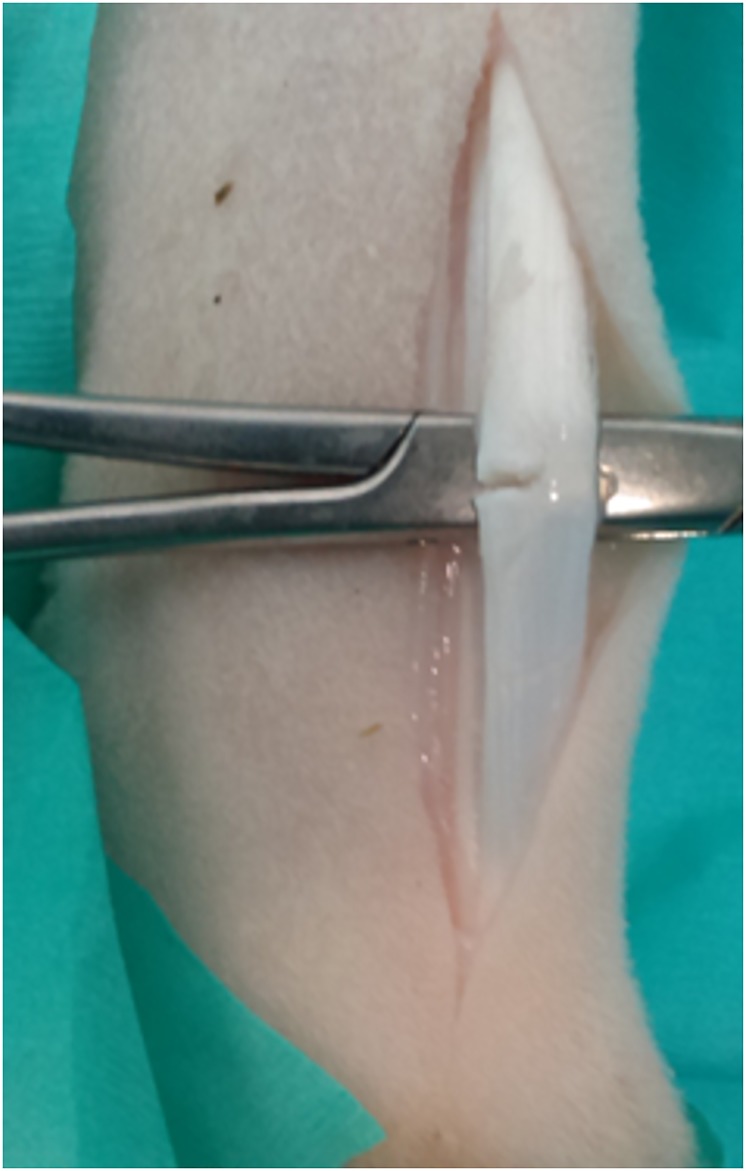
Surgical technique. The superficial digital flexor tendon (SDFT) was isolated and elevated before a 50% hemi-transection was performed on the lateral side.

The entire metacarpus of one randomly assigned forelimb was clipped and the midpoint of the metacarpus was marked with indelible ink to determine the level of tendon transection. Following aseptic preparation, a 5cm longitudinal skin incision was made over the palmarolateral aspect of the metacarpus. The fascia surrounding the flexor tendons was incised and the SDFT isolated and elevated with haemostats. A partial transection of the lateral aspect of the SDFT, proportionate to 50% of the width of the tendon, was performed with a scalpel blade (Fig 1). The paratenon and subcutaneous tissues were closed with absorbable multifilament sutures [3–0 Vicryl (Polyglactin 910), Ethicon LLC, Puerto Rico, USA] and the skin was closed with absorbable monofilament sutures [3–0 PDS II (Polydioxanone), Ethicon LLC, Puerto Rico, USA]. A light bandage, consisting of sterile swabs [BSN Medical (Aust.) Pty Ltd, VIC, Australia] and cohesive bandage [Henry Schein Inc, NY, USA], was applied to the limb.

#### Post-operative period

All sheep recovered from anaesthesia uneventfully and were housed in groups, in bedded stalls for seven days. There were no postoperative complications and sheep were fully weight-bearing shortly after surgery. After seven days, bandages were removed and the sheep were returned to pasture where they were monitored daily.

### Sample collection

The sheep were euthanised eight weeks after surgery with an overdose of pentobarbitone sodium [Lethabarb, Virbac (Australia) Pty Ltd, NSW, Australia]. Immediately following euthanasia the forelimbs of all sheep were clipped and cleaned with 70% isopropyl alcohol. An incision was made longitudinally along the palmar aspect of the forelimb from the accessory carpal bone proximally to the metacarpophalangeal joint distally. The SDFT and DDFT were exposed, isolated and transected proximally at the level of the accessory carpal bone and distally at the level of the metacarpophalangeal joint. The tendon was wrapped in a gauze swab moistened with sterile saline and placed into a sealed bag. The bagged samples were then placed on ice and processed within six hours of euthanasia.

**Fig 2:**
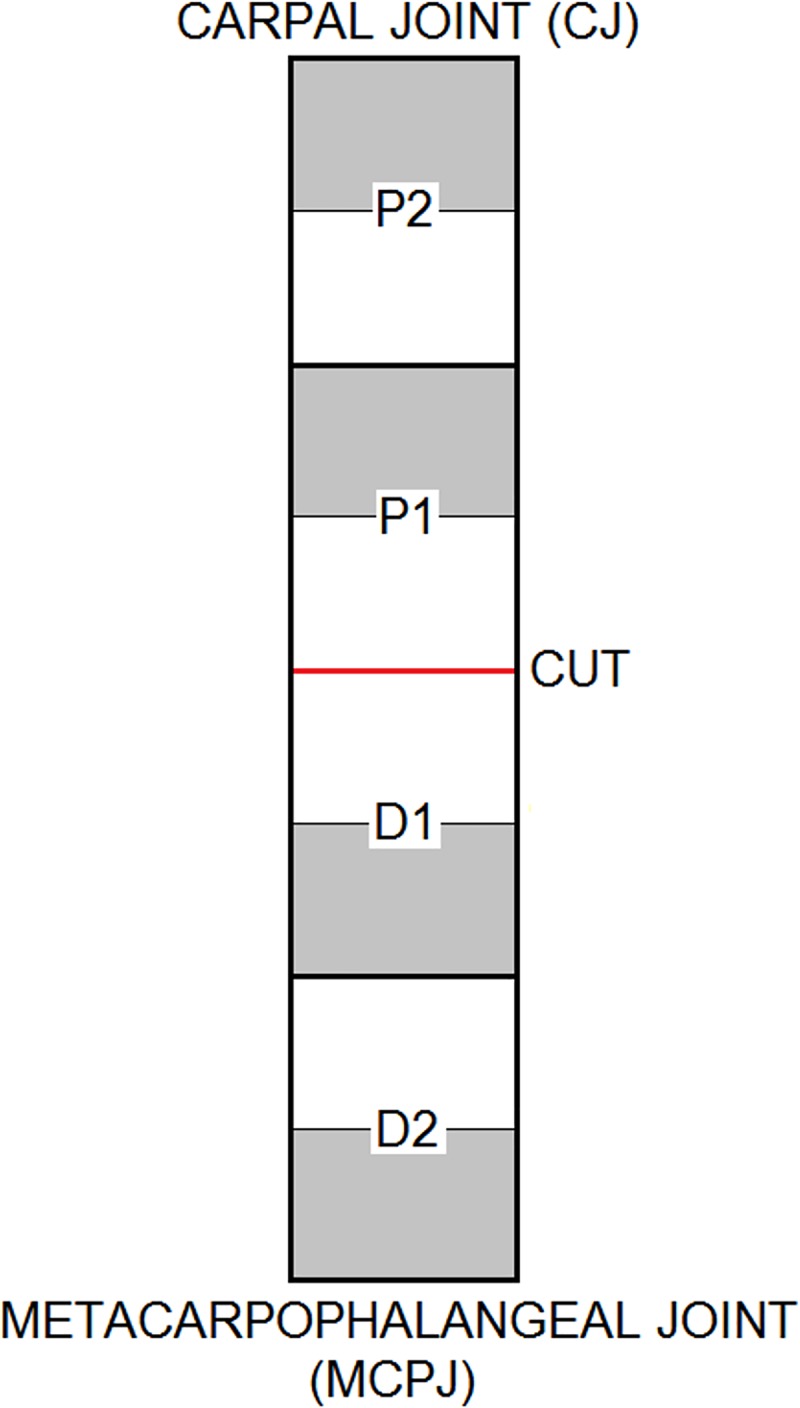
Sample collection diagram. The red line and “CUT” designate the level of either hemi-transection of the SDFT or complete transection of the extensor tendons. “P” and “D” indicate either proximal or distal to the level of the transection and the numbers indicate the regional division of the tendon. The white regions were samples for histology and the grey regions for gene expression analysis.

To obtain samples for histology and gene expression analysis, the paratenon was removed and each tendon was then divided regionally, with two equal regions above and below the level of the transection. These sections were split transversely into two sections, one for histology and one for gene expression analysis (Fig 2). The proximal end of each section was marked with 5% Alcian blue dye to orientate the samples for histopathology.

Samples for gene expression analysis were trimmed of any epitenon and frozen in liquid nitrogen before being stored at -80^o^ C. Histology samples were placed in 10% (v/v) neutral buffered formalin.

#### Histology

Histology samples were processed as previously described [1, 18]. Briefly, tendon samples were fixed for 48 hours in 10% (v/v) neutral buffered formalin then dehydrated in 70% ethanol (v/v) for 36 hours. Specimens were cleared in methyl benzoate for 72 hours prior to infiltration with 1% (w/v) celloidin and 1.5% (w/v) tricresyl phosphate in methyl benzoate for 72 hours then infiltrated with 5% (w/v) celloidin with 1.5% (w/v) tricresyl phosphate in methyl benzoate for three weeks. Samples were rinsed with chloroform (three changes) and infiltrated with paraffin wax (eight changes over four days) before being embedded in paraffin blocks. Paraffin blocks were softened for six hours with 5% (v/v) formic acid, 45% (v/v) ethanol and 50% (v/v) glycerol on a cold plate then rinsed with cold water prior to cutting. Five micron sections were then cut using a rotary microtome (Leica RM2255, Leica Microsystems, Germany) with Feather blades N35 (Arthur Bailey Surgico, NSW, Australia). Serial sections from each region were stained using standard methods with haematoxylin and eosin (H & E), picrosirius red (PSR) or toluidine blue (TB) [29].

Histology slides were scored independently by two observers (MS and AT), who were blinded with regard to treatment group, according to the previously described grading system [18, 29]. Observers were shown representative images for each grade for reference, prior to commencing scoring. Scoring of histological parameters was based on features of the tendon substance; assessment of the epitenon was not included. Slides stained with H & E were scored for the following parameters: intrafascicular cellularity, intrafascicular tenocyte morphology, interfascicular inflammatory cell infiltration, and vascularity. Toluidine blue stained slides were scored for proteoglycan accumulation whilst PSR slides, viewed under optimised polarised light, were scored for collagen fibre alignment. This qualitative linear polarised light microscopy technique is a versatile tool for subjectively assessing global collagen organisation and fibre alignment, however, rather than directly visualising the collagen fibres, orientation is inferred from the optical characteristics of the sample [31, 32]. This technique has been utilised to assess collagen fibre alignment in several models of large animal tendinopathy [1, 18, 26, 27].

All categories were assigned a score from 0–3 (except for intrafascicular cellularity), where 0 equates to a normal appearance and 3 indicates a highly pathological tendon (S1 Table). Cellularity was graded on a scale of 0–4, where 0 equates to numbers of cells observed in normal tendon, grades 1–3 were for increasing numbers of cells and an additional grade of 4 was also included for any potential decrease in cell numbers. A total histopathology score (0–15) was calculated from the sum of all ordinal scores except for proteoglycan score. An increase in proteoglycan accumulation is a normal finding in the insertional regions of tendons and may falsely elevate the total histopathology score; so, proteoglycan score was excluded as a component of the total histopathology score. Tendon sections with higher total histopathology scores indicated a more pathological appearance. Images of representative sections were obtained using a light microscope (polarised for PSR sections) and digital camera with Image Manager software (Leica Microsystems, Germany).

#### Gene expression analysis

Tendon samples were processed within weeks of sample collection as previously described [1, 18]. Briefly, frozen tendon sections were weighed and trimmed over dry ice (to between 80–15μg) before being pulverised into powder in a Dismembrator (Braun, Melsungen, Germany). RNA extraction was performed, following manufacturer instructions, with TRIzol reagent (Invitrogen, VIC, Australia), chloroform and RNeasy MiniKits (Qiagen, VIC, Australia) with an on-column DNase step (RNase-Free DNase Set; Qiagen) to eliminate any genomic DNA contamination. A Nanodrop spectrophotometer (Thermo-Fisher Scientific, Australia) was used to quantify RNA concentration and assess sample quality. A no-reverse transcription (no-RT) real time polymerase chain reaction (qPCR) step was performed to confirm RNA purity and check for any contaminant genomic DNA. RNA (1μg) from each sample was reverse transcribed into complementary DNA (cDNA) in a reaction volume of 50uL for three hours at 37°c using RT kits (GoScript from Promega) following manufacturer instructions except random pentadecamers (Sigma Genosys, NSW, Australia) instead of hexamers were used. Real-time polymerase chain reaction (PCR) was performed in a RotorGene 6000 analyser (Corbett Life Science, NSW, Australia) using Immomix (Bioline) and SYBRGreen II (Cambrex). Twenty genes were analysed and specific primers (Sigma Genosys, NSW, Australia) were used as previously described (S2 Table)[29]. This panel of 20 genes was selected as it encompasses the major fibrillar collagens and proteoglycans within the ECM as well as the metalloproteinases and inhibitors that are involved in the metabolism and repair of tendon matrix. Significant changes in the genes have previously been reported in large animal models of tendinopathy and disease [1, 18, 29].

Standard curves (four fold dilutions of ovine tendon cDNA) were included in each run and expression (relative fluorescence units) determined for each gene using RotorGene real-time qPCR software (Corbett Life Sciences, NSW, Australia). The threshold cycle (Ct–the cycle at which the fluorescence in the tube increases significantly above background) for each gene was determined and subsequently converted to a relative fluorescence unit (RFU) by interpolation of a standard curve by RotorGene qPCR software (Corbett Life Sciences, NSW, Australia), as previously validated [33]. This automated method has advantages of detecting efficiency and high cycle inhibition whilst providing similar values to both the delta-delta Ct and the Pfaffl methods [33]. Melt curves were produced after each qPCR run to verify a single identical gene-specific amplification product.

Gene expression data were normalised to total RNA rather than the mRNA of a specific reference gene. Recent literature has raised concerns regarding the selection and use of housekeeping genes, suggesting that there are none which can be used in every tissue or in particular disease states [34–37]. This is especially the case in musculoskeletal tissues that experience alterations in load after injury, where stable expression of generally used reference genes could not be identified [38]. Due to these concerns and our previous experience [1, 18], the fold difference of expression was calculated by dividing each sample RFU by the baseline non-operated control value of each gene for each region of the tendon.

#### Statistical analysis and data visualisation

Statistical analysis was performed using Stata IC version 14. Histopathology data were analysed for differences between groups and regions using a Kruskal-Wallis analysis, followed by a Mann-Whitney U test. A Benjamini-Hochberg post-hoc correction was applied. Significance was determined to be *P*<0.028 for these regression results after correction.

Prior to analysis, a logarithmic transformation was performed on the raw gene expression data as this was not normally distributed. Mixed regression modelling of each gene was carried out with data clustered by sheep and limb. Where differences were identified in the mixed model, univariate analysis was carried out to determine differences between groups (TxSDFT, TxEXT and NOC) using a Kruskal-Wallis analysis, followed by a Mann-Whitney U test where differences were observed. The fold change between control and treatment groups in each region was calculated for each gene using the equation: relative fluorescent units of each region/mean relative fluorescent units of the corresponding region of the controls.

Associations between histopathology scores and gene expression were determined by generating partial correlation coefficients using Kendall’s tau-b analysis with the subprogram “parttau” (James Fiedler and Alan Feiveson, Johnson Space Centre, Houston, Tx, USA) within Stata to correct for confounders (surgery and region). A Benjamini-Hochberg post-hoc correction was applied to the P values of each association.

Results are graphically represented as box plots with hinges at the 25^th^ and 75^th^ percentile and medians marked with a black line. Data are displayed with the tendon region along the x-axis, with proximal to the left and distal to the right. “CUT” designates the level at which either complete transection or partial transection of regional tendons was performed. Data are presented as relative fluorescent units (RFU) per μg of RNA.

## Results

### Gross morphology

All tendons were easily removed from their surrounding sheaths and no visible adhesions were evident between the paratenon and surrounding tissue. Control tendons were off-white in colour, firm and resilient in texture and were difficult to cut transversely with a scalpel. The middle of the partially transected SDFT was visibly thickened and pink in colour, as previously described [1]. No gross abnormalities were noted in the DDFT after partial transection of the SDFT or the SDFT and DDFT after complete transection of the extensor tendons. The epitenon of all tendon samples could easily be removed from gene expression samples.

### Histopathology

**Fig 3:**
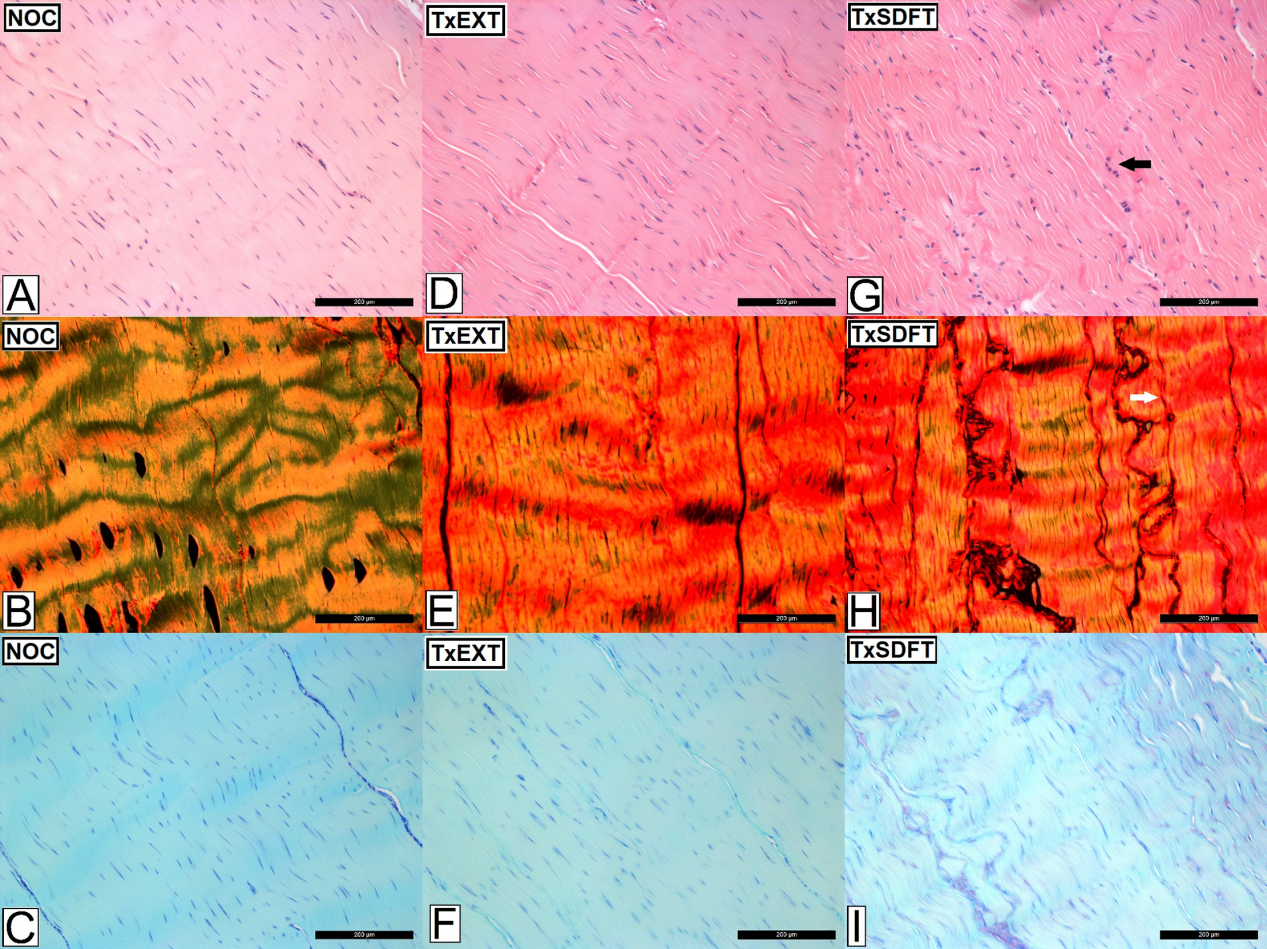
Histological appearance of the deep digital flexor tendon (DDFT) after complete transection of extensor tendons and partial transection of the superficial digital flexor tendon (SDFT). Images A-C represents the non-operated controls (NOC), images D-F represent the DDFT following complete transection of the extensor tendons (TxEXT) and images G-I represent the DDFT following partial transection of the SDFT (TxSDFT). Images A,D and G are stained with haemotoxylin and eosin (H&E), images B, E and H are stained with picrosirius red (PR) viewed under polarised light and images C, F and I are stained with toluidine blue (TB). Increased cellularity and cellular rounding are represented by the black arrow. The white arrow represents a loss of normal coarse crimp and cocrimping indicative of alteration in collagen fibre alignment. The scale bar indicates 200μm.

**Fig 4:**
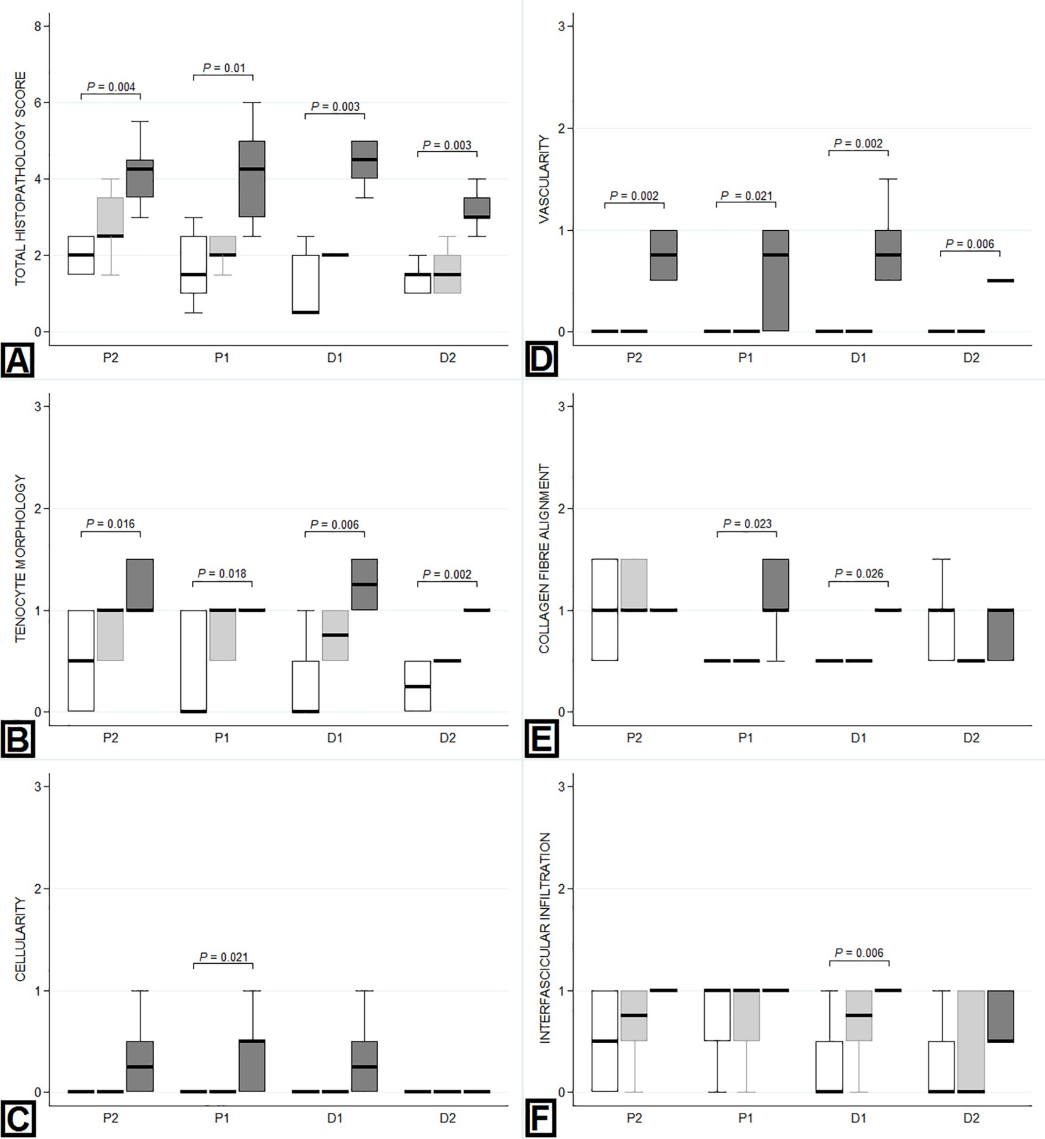
Regional variation in histopathology scores within the DDFT. Tendons within the non-operated control (NOC) group are represented by the white box (first box), the transected extensor tendon (TxEXT) group by the light grey box (second box) and the partially transected SDFT (TxSDFT) group by the dark grey box (third box). The mean numerical score for each histologic parameter is displayed on the Y-axis. The X-axis represents the regions sampled, with P2 being closest to the carpus and D2 being closest to the metacarpophalangeal joint. The level of transection in either the extensor tendons or SDFT would lie between P1 and D1. P-values for significant differences (*P*<0.05) between treatment groups are indicated on the graph. A: Total histopathology score. B: Tenocyte morphology. C: Cellularity. D: Vascularity. E: Collagen fibre alignment. F: Interfascicular cellular infiltration.

Representative images of H&E, PSR and TB-stained sections of tendon are presented in Fig 3. All histological parameters, except proteoglycan score, were significantly increased in the DDFT compared to controls following partial transection of the SDFT (Table 1). Total histopathology score (*P*<0.023), vascularity (*P*<0.021) and cell morphology (*P*<0.019) scores were significantly higher in the DDFT following partial transection of the SDFT compared to the control tendons in all regions (Fig 4). Scores for collagen fibre alignment were elevated adjacent to the site of injury (proximal and distal; *P*<0.026). Cellularity score was increased (*P*<0.008) immediately proximal to the site of injury, whilst cellular infiltration was increased (*P*<0.014) immediately distal to the injury site. Proteoglycan scores were not elevated in the DDFT following partial transection of the SDFT compared to control tendons.

**Table 1:** Comparison of histology scores between treatment groups (non-operated control, transected extensor tendons and partially transected superficial digital flexor tendon).

Histopathology Variable	Region	Surgery Comparison	KW (p-value)	MWU (p-value)
Proteoglycan content	P2	All	0.034	
		NOC vs TxEXT		>0.99
		TxSDFT vs NOC		0.058
	P1	All	0.37	
	D1	All	>0.99	
	D2	All	0.14	
Cellularity	P2	All	0.034	
	P1	All	**0.008**	
		NOC vs TxEXT		>0.99
		TxSDFT > NOC		**0.021**
	D1	All	0.034	
	D2	All	0.59	
Tenocyte Morphology	P2	All	**0.022**	
		NOC vs TxEXT		0.16
		TxSDFT > NOC		**0.016**
	P1	All	**0.024**	
		NOC vs TxEXT		0.081
		TxSDFT > NOC		**0.018**
	D1	All	**0.004**	
		NOC vs TxEXT		0.042
		TxSDFT > NOC		**0.006**
	D2	All	**0.001**	
		NOC vs TxEXT		0.043
		TxSDFT > NOC		**0.002**
Vascularity	P2	All	**<0.001**	
		NOC vs TxEXT		0.32
		TxSDFT > NOC		**0.002**
	P1	All	**0.008**	
		NOC vs TxEXT		>0.99
		TxSDFT > NOC		**0.021**
	D1	All	**<0.001**	
		NOC vs TxEXT		>0.99
		TxSDFT > NOC		**0.002**
	D2	All	**0.002**	
		NOC vs TxEXT		>0.99
		TxSDFT > NOC		**0.006**
Interfascicular infiltration	P2	All	0.32	
	P1	All	0.71	
	D1	All	**0.014**	
		NOC vs TxEXT		0.11
		TxSDFT > NOC		**0.006**
	D2	All	0.16	
Collagen fibre alignment	P2	All	0.72	
	P1	All	**0.006**	
		NOC vs TxEXT		0.32
		TxSDFT > NOC		**0.023**
	D1	All	**0.024**	
		NOC vs TxEXT		>0.99
		TxSDFT > NOC		**0.026**
	D2	All	0.15	
Total Histopathology Score	P2	All	**0.004**	
		NOC vs TxEXT		0.094
		TxSDFT > NOC		**0.004**
	P1	All	**0.006**	
		NOC vs TxEXT		0.33
		TxSDFT > NOC		**0.01**
	D1	All	**0.002**	
		NOC vs TxEXT		0.11
		TxSDFT > NOC		**0.003**
	D2	All	**0.003**	
		NOC vs TxEXT		0.67
		TxSDFT > NOC		**0.003**

Significance was set at *P*<0.028 (bold) after Benjamini-Hochberg correction. KW = Kruskal-Wallis analysis; MWU = Mann-Whitney U analysis; NOC = non-operated control; TxEXT = completely transected extensor tendons; TxSDFT = partially transected superficial digital flexor tendon (SDFT). The column for ‘Region’ refers to the regions sampled as per Fig 2; with P2 being closest to the carpus and D2 being closest to the metacarpophalangeal joint. The level of transection in either the extensor tendons or SDFT would lie between P1 and D1.

**Fig 5:**
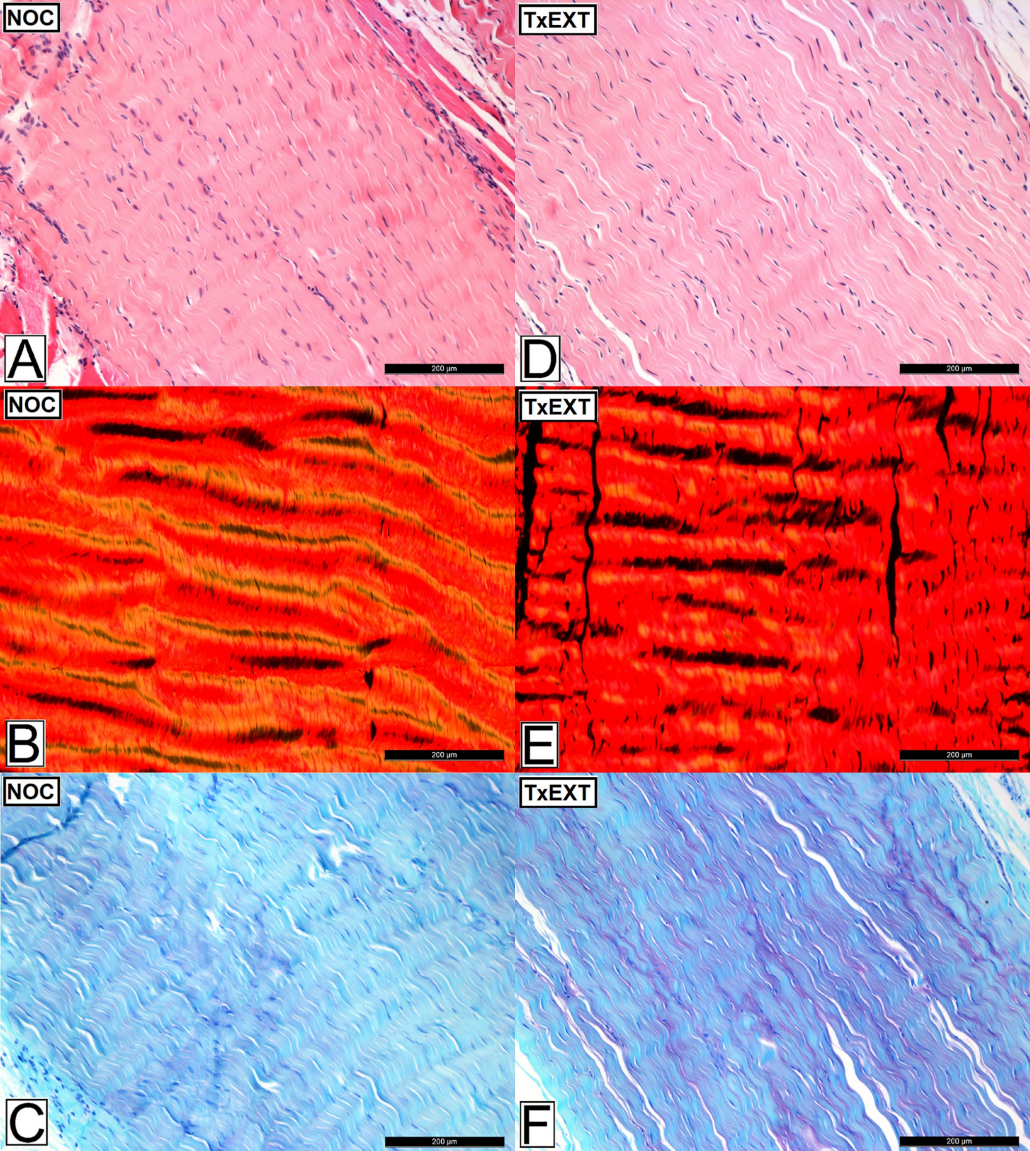
Histological appearance of the superficial digital flexor tendon (SDFT) after complete transection of extensor tendons. Images A-C represent the non-operated controls (NOC), images D-F represent the SDFT following complete transection of the extensor tendons (TxEXT). Images A and D are stained with haemotoxylin and eosin (H&E), images B and E are stained with picrosirius red (PR) viewed under polarised light and images C and F stained with toluidine blue (TB). The scale bar indicates 200μm.

There were no differences for all histological parameters in either the SDFT (S3 Table) or DDFT compared to control tendons after complete transection of the extensor tendons (Figs 3 and 5), except for a higher proteoglycan score in the most distal region of the control SDFT group (*P*<0.019) compared with the transected extensor group.

### Gene expression

We investigated the potential underlying molecular mechanisms initiating pathological changes in the subject tendons following either partial transection of the SDFT or complete transection of the extensor tendons. This was done by comparing the expression of key structural proteins and matrix remodelling enzymes and their inhibitors. Mean RNA yields in the DDFT for the different experimental groups, controls, partial transection of SDFT and complete transection of extensor tendons were 59.2, 73.7 and 71.9 ng/mg; respectively. Mean RNA yields in the SDFT were 74.0 ng/mg for the control group and 75.5 ng/mg for complete transection of the extensor tendons. A summary for the results of the mixed regression analysis for gene expression is presented in Tables 2 and 3.

**Table 2:** Effects of surgery and spatial position on matrix gene expression by mixed regression modelling of log-transformed expression data for each gene within the DDFT.

		Covariates		
Gene	P for model	Effect of Transection	Effect of proximity to carpus (C) or metacarpo-phalangeal joint (MCP)	Effect of distance from mid-metacarpus (lesion site)
Controls only				
COL3	**0.002**		nd (0.906)	**Increase (<0.001)**
ACAN	0.115		**MCP > C (0.042)**	nd (0.651)
VCAN	**<0.001**		nd (0.214)	**Increase (<0.001)**
ADAMTS4	0.077		nd (0.525)	**Decrease (0.030)**
ACTA-2	**0.012**		**MCP > C (0.021)**	nd (0.062)
Controls and operated				
COL3	**0.001**	**Increase (0.003)**	nd (0.291)	**Increase (0.011)**
VCAN	**<0.001**	**Increase (0.026)**	nd (0.401)	**Increase (<0.001)**
BGN	**0.003**	**Increase (0.004)**	nd (0.097)	nd (0.094)
LUM	**<0.001**	**Increase (<0.001)**	nd (0.585)	nd (0.951)
MMP1	0.170	**Increase (0.050)**	nd (0.782)	nd (0.294)
MMP9	**0.005**	nd (0.35)	**MCP > C (0.001)**	nd (0.412)
ADAMTS4	0.106	**Increase (0.033)**	nd (0.539)	nd (0.278)

Data was clustered by sheep and proximity to carpus (C) or metacarpophalangeal joint (MCP). Results are given for significant models without transection (n = 24) and from all models with transection (n = 72). nd = no difference; C = carpus; MCP = metacarpophalangeal joint.

**Table 3:** Effects of surgery and spatial position on matrix gene expression by mixed regression modelling of log-transformed expression data for each gene within the SDFT.

		Covariates		
Gene	P for model	Effect of Transection	Effect of proximity to carpus (C) or metacarpo-phalangeal joint (MCP)	Effect of distance from mid-metacarpus (lesion site)
Controls only				
COL2	**0.021**		**MCP > C (0.006)**	0.659
VCAN	**0.009**		**C > MCP (0.023)**	**Increase (0.041)**
ADAMTS5	0.063		**MCP > C (0.030)**	0.555
GAPDH	**0.048**		0.682	**Decrease (0.015)**
Controls and operated				
COL1	**0.049**	0.174	**MCP > C (0.017)**	0.603
COL2	**0.002**	0.162	**MCP > C (<0.001)**	0.532
ACAN	**0.004**	0.254	**MCP > C (0.001)**	0.644
VCAN	0.071	0.537	**C > MCP (0.012)**	0.525
BGN	**0.012**	0.332	**MCP > C (0.006)**	0.109
DCN	0.069	0.285	**MCP > C (0.029)**	0.248
ADAMTS5	**<0.001**	0.491	**MCP > C (<0.001)**	**Decrease (0.036)**
TIMP2	**0.020**	0.449	**MCP > C (0.011)**	0.097
GAPDH	**<0.001**	0.991	**MCP > C (0.001)**	**Decrease (0.005)**

Data was clustered by sheep and proximity to carpus (C) or metacarpophalangeal joint (MCP). Results are given for significant models without transection (n = 24) and from all models with transection (n = 48). nd = no difference; C = carpus; MCP = metacarpophalangeal joint.

**Fig 6:**
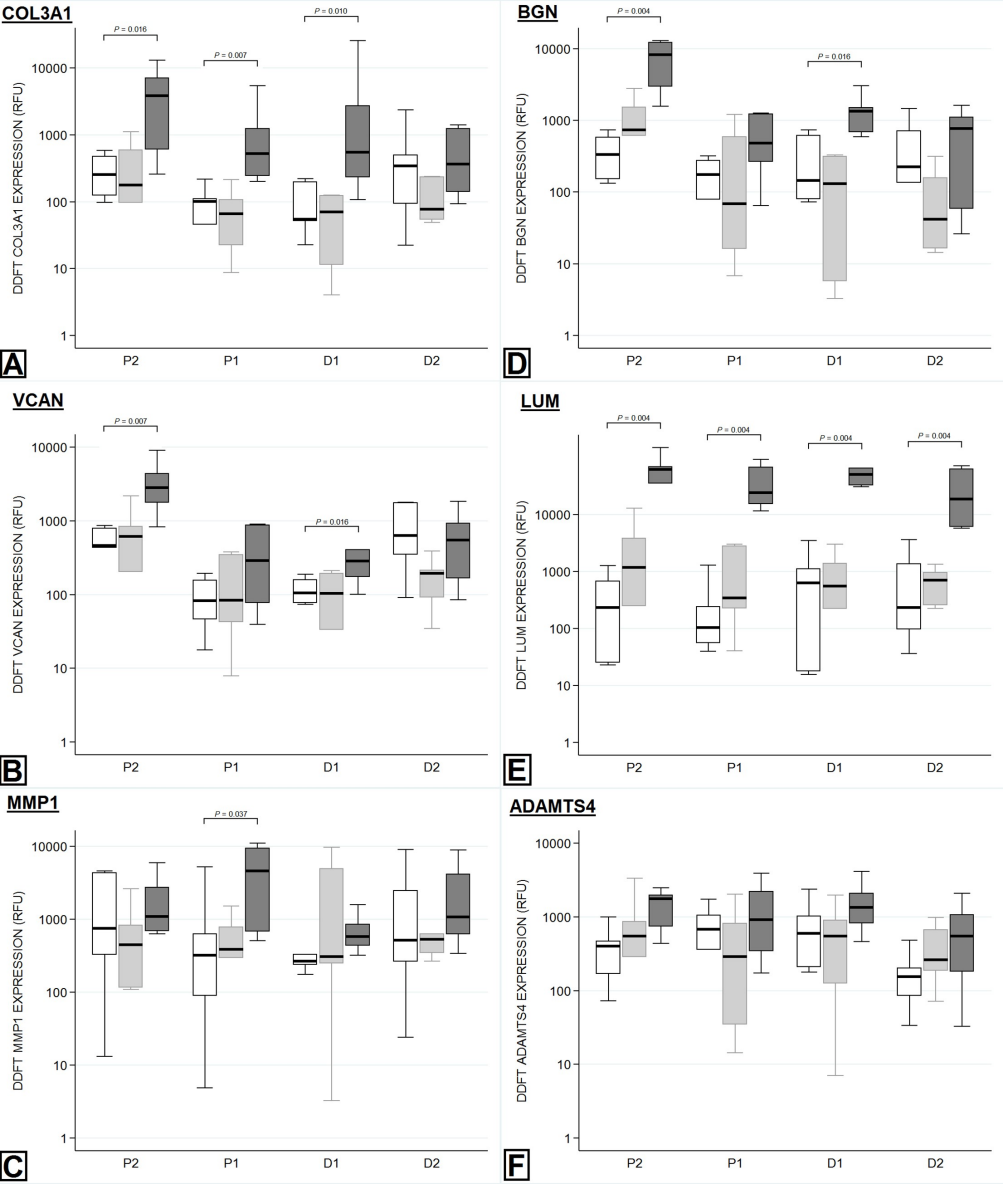
Regional variation in gene expression within the DDFT. Tendons within the non-operated control (NOC) group are represented by the white box (first box), the transected extensor tendon (TxEXT) group by the light grey box (second box) and the partially transected SDFT (TxSDFT) group by the dark grey box (third box). The relative fluorescent unit (RFU) per μg of RNA is displayed on the Y-axis. The X-axis represents the regions sampled, with P2 being closest to the carpus and D2 being closest to the metacarpophalangeal joint. The level of transection in either the extensor tendons or SDFT would lie between P1 and D1. P-values for significant differences (*P*<0.05) between treatment groups are indicated on each graph. A: Collagen III. B: Versican. C: MMP1. D: Bigylcan. E: Lumican. F: ADAMTS4.

Partial transection of the SDFT resulted in gene expression changes throughout the entire length of the DDFT, although some changes were localised with respect to proximity of the lesion. Collagen III (*COL3A1*) expression was increased adjacent to the injury (proximal and distal) and at the proximal extent of the tendon (up to 49.52 fold; P<0.016 for all regions). Expression of biglycan (*BGN*) and versican (*VCAN*) was increased just distal to the site of injury and at the proximal extent (up to 20.79 fold; P<0.025 for all regions). Lumican (*LUM*) expression was markedly increased throughout the entire tendon (up to 190.09 fold; P<0.004 for all regions) (Table 4). Expression of *MMP1* was increased just proximal to the level of the injury (4.69 fold; P = 0.037). These differences are represented graphically in Fig 6.

**Table 4:** Gene expression differences within the DDFT between non-operated control (NOC), transected extensor tendon (TxEXT) and partially transected SDFT (TxSDFT) groups.

Gene	Region	KW (p-value)	NOC vs Tx Ext	MWU (p-value)	NOC vs Tx SDFT	MWU (p-value)
COL3	P2	**0.019**	nd	0.749	**TxSDFT > NOC; 16.2 fold**	**0.016**
	P1	**0.006**	nd	0.522	**TxSDFT > NOC; 14.4 fold**	**0.007**
	D1	**0.010**	nd	0.522	**TxSDFT > NOC; 49.5 fold**	**0.010**
	D2	0.209				
VCAN	P2	**0.013**	nd	0.631	**TxSDFT > NOC; 7.16 fold**	**0.007**
	P1	0.281				
	D1	**0.040**	nd	0.749	**TxSDFT > NOC; 7.10 fold**	**0.025**
	D2	0.140				
BGN	P2	**0.002**	nd	0.109	**TxSDFT > NOC; 20.8 fold**	**0.004**
	P1	0.144				
	D1	**0.007**	nd	0.423	**TxSDFT > NOC; 4.71 fold**	**0.016**
	D2	0.130				
LUM	P2	**0.004**	nd	0.337	**TxSDFT > NOC; 142 fold**	**0.004**
	P1	**0.002**	nd	0.150	**TxSDFT > NOC; 131 fold**	**0.004**
	D1	**0.003**	nd	0.873	**TxSDFT > NOC; 190 fold**	**0.004**
	D2	**0.003**	nd	0.522	**TxSDFT > NOC; 33.9 fold**	**0.004**
MMP-1	P2	0.203				
	P1	**0.039**	nd	0.631	**TxSDFT > NOC; 4.69 fold**	**0.037**
	D1	0.249				
	D2	0.402				
ADAMTS4	P2	0.097				
	P1	0.386				
	D1	0.220				
	D2	0.250				

Mean fold changes for each region were calculated by dividing the relative fluorescent unit (RFU) value of the TxEXT and TxSDFT groups by the mean RFU of each region of the NOC group. P values are presented for each comparison. KW = Kruskal-Wallis analysis; MWU = Mann-Whitney U analysis; NOC = non-operated control; TxEXT = completely transected extensor tendons; TxSDFT = partially transected superficial digital flexor tendon (SDFT); nd = no difference. The column for ‘Region’ refers to the regions sampled as per Fig 2; with P2 being closest to the carpus and D2 being closest to the metacarpophalangeal joint. The level of transection in either the extensor tendons or SDFT would lie between P1 and D1.

Complete transection of the extensor tendons had no effect on the gene expression of any gene tested in either the SDFT (Table 3) or the DDFT (Table 4).

### Association studies

Interfascicular cellular infiltration was the only histopathology parameter assessed within the DDFT after partial transection of the SDFT that correlated with the gene expression results after Benjamini-Hochberg correction (significance set at *P*<0.005). Cellular infiltration correlated positively with increased production of *BGN* (0.434; *P*<0.001), *TIMP3* (0.395; *P* = 0.003) and *MMP2* (0.401; *P* = 0.005) and negatively with *MMP1* (-0.390; *P* = 0.003).

There were multiple positive and negative correlations between gene expression and histopathological parameters in both the SDFT and DDFT after complete transection of the extensor apparatus. However, since no significant differences between control and treated groups were observed and there was no apparent effect of extensor transection observed in the multi-variate model it is likely that these correlations are not related to the treatment.

## Discussion

It has recently been identified that there are widespread histopathological changes in the equine and ovine SDFT after focal surgical injury [1, 18]. From the results of this study, it has now been demonstrated that the DDFT sustains a mild tendinopathy following surgical injury to the SDFT and similar to previous findings [1, 18], these changes are not focal but in fact widespread throughout the entire tendon. Aspects of the tendinopathic changes observed in the SDFT, such as cellularity, collagen fibre malalignment and vascularity, did not appear to resolve over time and the authors considered that this pathology is likely to persist in the long term and have implications on the structure and function of the tendon [1, 18]. If these histopathologic changes within the SDFT do persist for the long term, the combined effect of pathology within both the SDFT and DDFT may contribute to the high recurrence of re-injury and the poor prognosis for return to athletic performance observed in equine athletes.

The development of chondroid metaplasia is a consistent feature of tendinopathy in both clinical and experimental studies [39–43]. A similar phenomenon was identified in this study within the DDFT with aspects of a chondroid phenotype, such as increased cellular rounding and cellularity, observed eight weeks after partial transection of the SDFT. However, in contrast to previous equine and ovine models of tendinopathy [1, 18, 29], increases in GAG content (proteoglycan scores) or increased gene expression of *ACAN* and *COL2A1* were not apparent in the DDFT. It is possible that sampling at eight weeks was too early to detect these changes, given that it is currently unclear when these changes first appeared within the DDFT. Alternatively, the DDFT may not develop all the features of chondroid metaplasia that are observed in commonly injured tendons and thus the structural and biomechanical changes may not be enough to cause dysfunction and clinical disease.

The cause of these changes within the DDFT after injury to the SDFT is unknown. It is possible that these changes are due to changes in biomechanics. It has previously been shown that following injury of the SDFT in one forelimb, force through the DDFT in the lame limb exceeds force through the contralateral DDFT during the stance phase of the gait [44]. This would suggest that biomechanical overload of the DDFT within the injured limb as a compensatory mechanism after injury to the SDFT is more likely to cause the changes observed within the DDFT than unloading of the affected limb. However, it is also feasible that the pathological changes observed are due to the local effect of inflammation. Given that the regions immediately adjacent to the level of the transection, both proximal and distal, were the more severely affected areas, it is possible that part of these changes in the DDFT could be explained by the regional influx of inflammatory cells and mediators as a direct result of surgery.

Changes in gene expression, both increases and decreases, have been observed in both clinical and experimental studies of tendon injury in humans and horses [18, 43, 45]. In contrast, in the DDFT after partial transection of the SDFT, there were only increases in the expression of five of the 20 targeted genes. Expression of collagen III was significantly increased immediately adjacent to the level of the transection, proximal and distal, and at the proximal extent of the DDFT. Collagen III gene expression and protein levels have previously been shown to be upregulated in the initial phases of tendon injury; resulting in changes in collagen III: I ratio [18, 46–48]. A positive correlation between *COL3A1* expression and cellular morphology, cellular infiltration and collagen fibre malalignment has previously been observed within the SDFT following surgical injury [1]; however these associations were not observed in this study. Collagen III is a weaker collagen fibre produced in repairing tissues, whilst *COL3A1* gene expression is elevated within several regions of the DDFT, the lack of correlations between *COL3A1* expression and the histological parameters, especially collagen fibre alignment, at an eight week time point of sampling suggests that the tendinopathy within the DDFT is early and the reparative response is currently limited.

Lumican and *BGN* play roles in normal fibrillogenesis [49–51]; however, recently they have all been associated with collagen fibre malalignment in healing tissues that result in detrimental regional biomechanics and ultimately poor outcomes [18]. Lumican has further been implicated in the development of pathological fibrosis in multiple tissue types [52, 53]. The accumulation of GAGs, specifically large aggregating proteoglycans aggrecan and versican, in injured SDFTs has also been shown to cause a reduction in modulus and ultimate tensile strength [54]. Positive correlations between increases in *LUM*, *BGN* and *VCAN* gene expression and increased proteoglycan score have previously been described in the ovine SDFT after surgical injury [1]. In contrast, this study demonstrated regional increases in gene expression of *LUM*, *BGN* and *VCAN* without a concomitant increase in proteoglycan score. Whilst there are inherent risks in comparing between studies and tissue types, the fold change increase in gene expression of these proteoglycans were much higher within the DDFT in this study than the fold change increases in these genes within the SDFT after partial transection of the SDFT at eight weeks in the previous study (up to 190 fold vs up to 13.9 fold) [1]. It is also worth noting that the exact timing of injury within the DDFT is unknown. As opposed to the surgical transection of the SDFT, the injury to the DDFT, whether biomechanical or inflammatory, likely occurs at a later time point. Therefore, it is possible that the time point of harvest, eight weeks after surgical injury of the SDFT, was too early to detect an increase in protein deposition and ultimately proteoglycan staining even though there was a significant increase in gene expression.

Biglycan has been observed to be markedly increased in chronically painful Achilles tendinopathy in humans [34]. As such, the positive correlation observed between cellular infiltration and increased expression of *BGN* within the DDFT following partial transection of the SDFT likely reflects the development of pathology within the DDFT. The negative association between cellular infiltration and *MMP1* expression suggests that infiltrating cells are not the primary source of collagenase within the DDFT.

This study demonstrates that the DDFT develops a mild subclinical tendinopathy, eight weeks after surgical injury of the adjacent SDFT in an ovine model. The altered structure and composition of the DDFT is likely to have a detrimental effect on its biomechanical properties and ultimately the healing of the adjacent injured SDFT. It is entirely feasible that this mild tendinopathy of the DDFT contributes to the high recurrence rates and poor outcomes of SDFT injuries. This finding raises questions about current diagnostic techniques as well as treatment and rehabilitation protocols. Ultrasound examination of tendons is the currently the most widely used technique for detection of injuries [55]; however, it has been shown to have a poor sensitivity [18]. Different techniques may need to be developed to accurately diagnose the affected tissue(s) but also to monitor the progression of healing during rehabilitation and ensure further injury does not occur.

The only significant difference observed within the flexor tendons following complete transection of the extensor tendons was a reduction in proteoglycan score in the most distal region of the SDFT of the transected compared with the control group (mean score of 0.67 vs 1, respectively). This distal section lies within the metacarpophalangeal compressive region of the SDFT as it traverses along the back of the metacarpophalangeal joint. The compressive region of tendons exhibits different properties compared with the tensional region; including increased proteoglycan staining, due to enhanced amounts of aggrecan, and rounded cellular morphology [16–18]. The overall reduction in proteoglycan staining in the TxEXT group was minor, still being in the “1” category with the NOC group, but this could suggest reduced compressive loading on this tendon region as a result of gait alterations with loss of extensor function. However, there was no concomitant change in expression of the key compression-associated proteoglycans (ACAN, BGN) or enzymes responsible for their turnover (ADAMTS4, ADAMTS5). The statistical difference likely reflects biological variation in the amount of proteoglycan staining between the individuals.

Following complete transection of the extensor tendons, horses commonly show a markedly abnormal gait that is characterised by the inability to extend the digit and ultimately means that they bear weight on the dorsal surface of the digit and fetlock [7, 56]. This study demonstrates that following complete transection of the extensor tendons there are minimal abnormal findings within either the SDFT or DDFT in an ovine model. This likely means that although some horses can display an abnormal gait for several weeks or months following injury this is unlikely to affect the structure or function of the flexor tendons moving forward. The observations observed in this study are consistent with the findings of several retrospective studies that demonstrate that complete transection of the extensor tendons has a good prognosis for return to full athletic function [57–60].

The suitability of a surgical transection model of tendinopathy to study naturally occurring disease is one limitation of this study. Several techniques have been developed to induce tendinopathy in laboratory and large animal models; however, there are limitations to each method. Overuse (forced treadmill running) does not explain tendinopathy in the sedentary [61], exogenous collagenases elicit an acute widespread inflammatory response and surgical transection is an artificial method expect for examining trauma induced degeneration [22, 62]. However, the utility of this model is supported by several studies, which have identified molecular and histologic changes in transected tendons typical of overuse tendinopathy [1, 18, 29].

Another limitation of this study is the use of only histology and gene expression to describe the changes that occur within adjacent tendons following injury to a separate tendon within the distal limb. Gene expression changes may not always be reflective of de novo synthesis of the protein and ultimately activity of the translated protein due to the possibility of post-translational modification and inhibition within the ECM [62]. However, as has previously been postulated, alterations in gene expression are likely to represent changes in extracellular environments with potential ensuing effects on tendon structure and function [62]. This suggestion has been reinforced by previous work that has identified increases in gene expression of proteoglycans, collagen III and metalloproteinases following surgical injury to tendon are reflected in increased immunohistochemical staining of these proteins [1, 18]. Although the techniques performed in this study are limited, the gene expression alterations and especially the histopathology changes observed, highlight the potential for injury of adjacent tendons following injury of a separate tendon. Further studies should focus on other analyses that were not performed in this study such as zymography to assess protease activity.”

Over-strain injuries within the SDFT are now recognised as a bilateral disease, with clinical signs observed in the more severely affected limb [63]. Whilst there were no preceding degenerative changes within the tendons prior to surgical injury, it would have been interesting to assess any changes within the contralateral flexor tendons, both the SDFT and DDFT, especially considering the findings of this study.

This study shows that the due to the complex relationships between tendons in the distal limb, a mild tendinopathy develops within the DDFT, eight weeks after surgical injury of the adjacent ovine SDFT. Further studies should aim to identify histologic, gene expression and biomechanical changes at more time points following injury and whether the observed changes in the DDFT resolve. Identifying whether this phenomenon occurs following naturally occurring SDFT tendinopathy will also be important. Ultimately, the changes observed in the DDFT may contribute to the poor outcomes of SDFT injuries and have lasting implications on the successful treatment and rehabilitation of the SDFT.

In conclusion, to the best of the authors’ knowledge, this is the first study that demonstrates mild subclinical tendinopathy of the DDFT, eight weeks after surgical injury of the adjacent ovine SDFT. Further studies should aim to identify histologic and gene expression changes at more time points following injury and whether the observed changes in the DDFT eventually resolve or become more severe. Identifying whether this phenomenon occurs in naturally occurring SDFT tendinopathy will also be important as the altered structure and composition of the DDFT is likely to have detrimental effects on its biomechanical properties. Ultimately, these changes may contribute to the high recurrence rates of SDFT injuries and have lasting implications on the successful treatment and rehabilitation of SDFT.

## Supporting information

S1 Table Histology scores and descriptions. Histology scoring parameters are shown. Total histopathology score was calculated from the sum of all scores except proteoglycan content. A higher score indicated a tendon with a more pathological appearance.
(DOCX)

S2 Table Analysed genes and relevant primers. Analysed genes (with standard abbreviations as used in the text), primer sequences, annealing temperatures and product sizes are shown.
(DOCX)

S3 Table Comparison of histology scores within the SDFT between non-operated control (NOC) and transected extensor tendons (TxEXT). Significance was set at *P*<0.05 (bold). MWU = Mann-Whitney U analysis; NOC = non-operated control; TxEXT = completely transected extensor tendons.
(DOCX)

S1 Spreadsheet Dataset with raw data for all gene expression and histology analyses. (XLSX)
