# Supplementary material for: Effects of tendon injury on uninjured regional tendons in the distal limb: An in-vivo study using an ovine tendinopathy model
Source: PLoS One. 2019 Apr 23;14(4):e0215830. doi: 10.1371/journal.pone.0215830 (PMC6478347; doi:10.1371/journal.pone.0215830)
Supplement: S1 Table — Histology scoring parameters are shown. Total histopathology score was calculated from the sum of all scores except proteoglycan content. A higher score indicated a tendon with a more pathological appearance. (DOCX) [file pone.0215830.s001.docx]

| Outcome measure | Stain | Score | Description |
| --- | --- | --- | --- |
| Cellularity | H&E | 0 | Normal |
|  |  | 1 | Mild increase |
|  |  | 2 | Moderate increase |
|  |  | 3 | Marked increase |
|  |  | 4 | Decrease |
| Tenocyte morphology | H&E | 0 | 75-100% tenocytes normal/spindle-shaped (0-25% tenocytes abnormal/rounded) |
|  |  | 1 | 50-75% tenocytes normal/spindle-shaped (25-50% tenocytes abnormal/rounded) |
|  |  | 2 | 25-50% tenocytes normal/spindle-shaped (50-75% tenocytes abnormal/rounded) |
|  |  | 3 | 0-25% tenocytes normal/spindle-shaped (75-100% tenocytes abnormal/rounded) |
| Vascularity | H&E | 0 | Normal |
|  |  | 1 | Mild increase |
|  |  | 2 | Moderate increase |
|  |  | 3 | Marked increase |
| Interfascicular infiltration | H&E | 0 | Normal |
|  |  | 1 | Mild increase |
|  |  | 2 | Moderate increase |
|  |  | 3 | Marked increase |
| Collagen fibre alignment | Picro-sirius red | 0 | 75-100% normal alignment |
|  |  | 1 | 50-75% normal alignment |
|  |  | 2 | 25-50% normal alignment |
|  |  | 3 | 0-25% normal alignment |
| Proteoglycan content | Toluidine blue | 0 | Normal |
|  |  | 1 | Mild increase |
|  |  | 2 | Moderate increase |
|  |  | 3 | Marked increase |
