# Supplementary material for: Effects of tendon injury on uninjured regional tendons in the distal limb: An in-vivo study using an ovine tendinopathy model
Source: PLoS One. 2019 Apr 23;14(4):e0215830. doi: 10.1371/journal.pone.0215830 (PMC6478347; doi:10.1371/journal.pone.0215830)
Supplement: S2 Table — Analysed genes (with standard abbreviations as used in the text), primer sequences, annealing temperatures and product sizes are shown. (DOCX) [file pone.0215830.s002.docx]

| **Target gene** | **Sequence 5' to 3'** | **Annealing temperature (°c)** | **Product size (bp)** |
| --- | --- | --- | --- |
| Collagen I (COL1A1) | F: ATC CCT GGA CAA CCT GGA CTT C R: TCA TCA TAG CCG TAA GAC AAC TGG | 57 | 107 |
| Collagen II (COL2A1) | F: TGA CCT GAC GCC CAT TCA TC R: TTT CCT GTC TCT GCC TTG ACC C | 55 | 154 |
| Collagen III (COL3A1) | F: GCA GGG AAC AAC TTG ATG GTG C R: AAT AGT GGG ATG AAG CAG AGC G | 55 | 144 |
| Aggrecan (ACAN) | F: TCA CCA TCC CCT GCT ACT TCA TC  R: TCT CCT TGG AAA TGC GGC TC | 58 | 105 |
| Versican (VCAN) | F: CAT CTC ACC AGT ATC CTG TCT CAC G R: AGT GTG CTG CCA TCA GTC CAA C | 55 | 128 |
| Biglycan (BGN) | F: TGA TTG AGA ACG GGA GCC TGA G R: TTT GGT GAT GTT GTT GGT GTG C | 56 | 143 |
| Fibromodulin (FMOD) | F: GCT CCA TCT TGA CCA CAA CCA G R: CCT TTC ATA GAA CTG CCC ACT TCC | 55 | 123 |
| Decorin (DCN) | F: CCA AAG TGC GAA AGT CTG TGT TC R: CAG CAA TGC GGA TGT AGG AGA G | 54 | 138 |
| Lumican (LUM) | F: TGG CTG ATA GTG GAG TTC CTG G R: GGT TTT CAT TGA CTG TCG GTA TGC | 50 | 105 |
| MMP1 | F: CAT TCT ACT GAC ATT GGG GCT CTG R: TGA GTG GGA TTT TGG GAA GGT C | 55 | 122 |
| MMP2 | F: TGC TAC CAC CTC CAA CTA CGA TG R: GTG CCA GTA TCA ATG TCA GGG G | 60 | 240 |
| MMP9 | F: AGG TGA ATC AGG TGG ACT ATG TGG R: AGA AAG GAA GGT GGG AAG AGA GG | 59 | 221 |
| MMP13 | F: CGT ATT GAT GCT GCC TAT G R: GCT CCA GAC TTG GTT TTC T | 58 | 216 |
| ADAMTS4 | F: AAC TCG AAG CAA TGC ACT GGT R: TGC CCG AAG CCA TTG TCT A | 60 | 149 |
| ADAMTS5 | F: GCA TTG ACG CAT CCA AAC CC R: CGT GGT AGG TCC AGC AAA CAG TTA C | 55 | 97 |
| TIMP1 | F: GGT TCA GTG CCT TGA GAG ATG C R: GGG ATA GAT GAG CAG GGA AAC AC | 57 | 265 |
| TIMP2 | F: ACT CTG GCA ACG ACA TCT ACG G R: TCT TCT TCT GGG TGG CAC TCA G | 57 | 261 |
| TIMP3 | F: CTT CCT TTG CCC TTC TCT ACC C R: TCT GGT CAA CCC AAG CAT CG | 57 | 286 |
| GAPDH | F: CCT GGA GAA ACC TGC CAA GTA TG R: GGT AGA AGA GTG AGT GTC GCT GTT G | 58 | 139 |
| Alpha actin-2 (ACTA-2) | F: CTT TCC AGA ACA CCA CCC AGA G R: ATT GTC ACA CAC CAA GGC GG | 55 | 118 |
