# Supplementary material for: Effects of tendon injury on uninjured regional tendons in the distal limb: An in-vivo study using an ovine tendinopathy model
Source: PLoS One. 2019 Apr 23;14(4):e0215830. doi: 10.1371/journal.pone.0215830 (PMC6478347; doi:10.1371/journal.pone.0215830)
Supplement: S3 Table — Significance was set at P<0.05 (bold). MWU = Mann-Whitney U analysis; NOC = non-operated control; TxEXT = completely transected extensor tendons. (DOCX) [file pone.0215830.s003.docx]

| **Histopathology Variable** | **Region** | **Surgery Comparison** | **MWU (p-value)** |
| --- | --- | --- | --- |
| Proteoglycan content | P2 | NOC vs TxEXT | 0.14 |
|  | P1 | NOC vs TxEXT | 0.39 |
|  | D1 | NOC vs TxEXT | 0.092 |
|  | D2 | **NOC > TxEXT** | **0.019** |
| Cellularity | P2 | NOC vs TxEXT | 0.32 |
|  | P1 | NOC vs TxEXT | 0.85 |
|  | D1 | NOC vs TxEXT | 0.5 |
|  | D2 | NOC vs TxEXT | 0.12 |
| Tenocyte Morphology | P2 | NOC vs TxEXT | 0.2 |
|  | P1 | NOC vs TxEXT | 0.057 |
|  | D1 | NOC vs TxEXT | 0.18 |
|  | D2 | NOC vs TxEXT | 0.86 |
| Vascularity | P2 | NOC vs TxEXT | 0.78 |
|  | P1 | NOC vs TxEXT | 0.67 |
|  | D1 | NOC vs TxEXT | 0.21 |
|  | D2 | NOC vs TxEXT | 0.79 |
| Interfascicular infiltration | P2 | NOC vs TxEXT | 0.12 |
|  | P1 | NOC vs TxEXT | 0.35 |
|  | D1 | NOC vs TxEXT | 0.8 |
|  | D2 | NOC vs TxEXT | 0.5 |
| Collagen fibre alignment | P2 | NOC vs TxEXT | 0.053 |
|  | P1 | NOC vs TxEXT | 0.14 |
|  | D1 | NOC vs TxEXT | 0.62 |
|  | D2 | NOC vs TxEXT | 0.16 |
| Total Histopathology Score | P2 | NOC vs TxEXT | 0.57 |
|  | P1 | NOC vs TxEXT | 0.26 |
|  | D1 | NOC vs TxEXT | 0.47 |
|  | D2 | NOC vs TxEXT | 0.94 |
